# Hierarchical improvement of regional tissue oxygenation after packed red blood cell transfusion

**DOI:** 10.1371/journal.pone.0271563

**Published:** 2022-07-20

**Authors:** Kiran Kumar Balegar V., Madhuka Jayawardhana, Andrew J. Martin, Philip de Chazal, Ralph Kay Heinrich Nanan

**Affiliations:** 1 Department of Neonatology, Nepean Hospital, Sydney Medical School Nepean, The University of Sydney, Sydney, NSW, Australia; 2 School of Electrical Engineering and the Charles Perkins Center, The University of Sydney, Sydney, NSW, Australia; 3 NHMRC Clinical Trials Centre, University of Sydney, Sydney, NSW, Australia; 4 School of Biomedical Engineering and the Charles Perkins Center, The University of Sydney, Sydney, NSW, Australia; 5 Sydney Medical School and Charles Perkins Center Nepean, The University of Sydney, Sydney, NSW, Australia; Universitat zu Lubeck, GERMANY

## Abstract

**Background:**

It is well established that counter-regulation to hypoxia follows a hierarchical pattern, with brain-sparing in preference to peripheral tissues. In contrast, it is unknown if the same hierarchical sequence applies to recovery from hypoxia after correction of anemia with packed red blood cell transfusion (PRBCT).

**Objective:**

To understand the chronology of cerebral and splanchnic tissue oxygenation resulting after correction of anemia by PRBCT in preterm infants using near-infrared spectroscopy (NIRS).

**Design:**

Prospective cohort study.

**Setting:**

Neonatal intensive care.

**Patients included:**

Haemodynamically stable infants: <32 weeks gestation, <37weeks postmenstrual age, <1500 grams birth weight; and ≥120 mL/kg/day feeds tolerated.

**Intervention:**

PRBCT at 15 mL/Kg over 4 hours.

**Main outcome measures:**

Transfusion-associated changes were determined by comparing the 4-hour mean pre-transfusion cerebral and splanchnic fractional tissue oxygen extraction (FTOEc0; FTOEs0) with hourly means during (FTOEc1-4; FTOEs1-4) and for 24 hours after PRBCT completion (FTOEc5-28; FTOEs5-28).

**Results:**

Of 30 enrolled infants, 14[46.7%] male; median[IQR] birth weight, 923[655–1064]g; gestation, 26.4[25.5–28.1]weeks; enrolment weight, 1549[1113–1882]g; and postmenstrual age, 33.6[32.4–35]weeks, 1 infant was excluded because of corrupted NIRS data. FTOEc significantly decreased during and for 24 hours after PRBCT (p < 0.001), indicating prompt improvement in cerebral oxygenation. In contrast, FTOEs showed no significant changes during and after PRBCT (p>0.05), indicating failure of improvement in splanchnic oxygenation.

**Conclusion:**

Improvement in regional oxygenation after PRBCT follows the same hierarchical pattern with a prompt improvement of cerebral but not splanchnic tissue oxygenation. We hypothesise that this hierarchical recovery may indicate continued splanchnic hypoxia in the immediate post-transfusion period and vulnerability to transfusion-associated necrotizing enterocolitis (TANEC). Our study provides a possible mechanistic underpinning for TANEC and warrants future randomised controlled studies to stratify its prevention.

## Introduction and objectives

It is well established that regional tissue hypoxia is heterogeneous, with splanchnic vasoconstriction occurring as an early physiologic response, diverting blood flow away from abdominal organs to the brain [1]. However, studies investigating the chronology of reversal of this process when the hypoxic drive is removed are lacking. This question is raised in the context of transfusion-associated necrotizing enterocolitis (TANEC). The occurrence of TANEC may indicate that splanchnic oxygen delivery continues to be suboptimal in the post-transfusion period.

Regional tissue oxygenation can be evaluated using near-infrared spectroscopy (NIRS). Near- infrared waves emitted by the NIRS sensor penetrate the underlying tissue, and travel along an arc at a depth of 1–2 cm. These waves are differentially absorbed by oxyhemoglobin and deoxyhemoglobin present in the microcirculation of the tissue within the travel pathway. The waves not detected by the photodetector of the NIRS sensor account for light absorbed by deoxyhemoglobin and oxyhemoglobin. The ratio of oxyhemoglobin vs. total hemoglobin is calculated as oxygen saturation of tissue hemoglobin (StO_2_) and represents the ‘weighted average’, of oxygen saturation of hemoglobin in arterial and venous compartments of the tissue located at a depth of 1–2 cm below the sensor” [2–14]. A NIRS sensor placed on the scalp measures StO_2_ in cerebral tissue 1–2 cm below the scalp. Likewise, a NIRS sensor placed on the abdomen measures the StO_2_ of splanchnic tissue. StO_2_ is, in turn, used to calculate the Fractional Tissue Oxygen Extraction (FTOE) [15–17]. FTOE indicates oxygen utilization efficiency of tissue, and is determined by oxygen consumption in the context of prevailing delivery. [FTOE = (oxygen consumption/oxygen delivery)]. FTOE is calculated using simultaneously measured arterial oxygenation using a pulse oximeter (SpO_2_) and StO_2_ using NIRS. FTOE = [(SpO_2_–StO_2_) X 100 / (SpO_2_)]. An increase in FTOE indicates that oxygen consumption is out of proportion to delivery [18, 19]. On the other hand, a decrease in FTOE indicates efficient tissue oxygen utilization with delivery being adequate for tissue demands. A trend rather than the absolute value of FTOE is more meaningful [15]. An increase in cerebral FTOE (FTOEc) has been associated with early brain injury in preterm infants [19].

One of the main concerns in using splanchnic NIRS is that the sampled tissue changes due to peristalsis, thereby producing wide fluctuations in mesenteric oxygenation and often swamping the signal of interest [20, 21]. To overcome this, longer periods of monitoring and trending the data using short data averaging time have been recommended [22]. Studies that simultaneously monitor cerebral and splanchnic oxygenation readings at a higher sampling frequency, for longer periods in relation to packed red blood cell transfusion (PRBCT) are paucal. The objective of the current study was to determine the chronology of changes in splanchnic and cerebral tissue oxygenation in premature infants receiving PRBCT using NIRS.

## Materials and methods

### Patient and public involvement

Patients and the Public were not involved in the design and conduct of the study.

The methodology was similar, and many of the participants were included in the previously published study [23]. This prospective observational study was conducted in the neonatal intensive care at Nepean Hospital, Australia, (Sep 2014- Nov 2016)—Eligible participants included: gestation <32 weeks; birth weight <1500 grams (g); postmenstrual age <37 weeks; tolerating enteral feed volume ≥120 millilitres/kilogram/day (mL/Kg/day); hemodynamically stable and receiving elective PRBCT to treat anemia of prematurity. Infants with necrotizing enterocolitis (NEC) (current/ previous); feed intolerance (clinician’s decision to withhold /withhold grading up of feeds for ≥12 hours); sepsis (clinician’s decision to commence antibiotics); PRBCT within 72 hours; patent ductus arteriosus (PDA)/its treatment within 72 hours and/or congenital gastrointestinal, complex cardiac/lethal anomalies were excluded. An infant was enrolled only once even when receiving multiple transfusions. For pragmatic reasons, enrolment occurred only when PRBCT was initiated during normal business hours (weekdays: 8 am-5 pm). Consecutive babies satisfying the above criteria were enrolled after a written informed parental consent. The study protocol was approved by the Hospital human research ethics committee (Approval number: Study 12/67—HREC/12/NEPEAN/148).

#### Study protocol

The study period extended from 4 hours before the PRBCT commencement until 24 hours after its completion. A calculated PRBCT volume of 15 mL/kg rounded up to the next whole number, infused over 4 hours, without Furosemide, was used. All babies received the same volume so as to standardize the dose of PRBCT. RBCs were non-irradiated, leucodepleted, Cytomegalovirus (CMV) negative, and stored in SAG-M additive solution (Sodium chloride, 8.77 g/L; Adenine, 0.169 g/L; Glucose, 9 g/L; Mannitol, 5.25 g/L). The decision to transfuse was made by the attending neonatologist independent of the study and was based on a combination of low hemoglobin (Hb) and clinical factors (e.g., respiratory support, increasing desaturations, poor growth, gestation, reticulocyte response, etc.). Feeding was continued throughout the study. Nature, frequency, and volume of feeding were determined by the clinical team independent of the study but remained the same before, during, and after PRBCT. For standardization, bolus feeds were given by a syringe pump at the rate of 120 mL/hour.

### Determination of fractional tissue oxygen extraction (FTOE)

During the study period, a continuous and concurrent evaluation of oxygen saturation of arterial hemoglobin (SpO_2_) (Radical-7® Pulse CO-Oximeter®, Masimo Corp. Irvine, CA), cerebral, and splanchnic StO_2_ (FORE-SIGHT® absolute cerebral oximeter, CASMED, Branford, Connecticut, 06405 USA) were made. For babies needing supplemental oxygen, the fraction of inspired oxygen concentration (FiO_2_) was adjusted to target SpO_2_ 91–95%. A neonatal NIRS sensor was placed over the temporal region of the head to obtain cerebral StO_2_ (StO_2_c) and a second sensor was placed on the abdomen just below the umbilicus [16, 24] to obtain simultaneous splanchnic StO_2_ (StO_2_s). Cerebral and splanchnic FTOE (FTOEc and FTOEs) were calculated using simultaneously measured StO_2_c, StO_2_s, and SpO_2_ as described previously. Skin integrity was closely monitored by lifting the sensors and inspecting the skin six hourly at the time of ’cares’ (handling of neonates for a nappy change, eye care, change of posture, etc.) The start and end times of the study, as well as various events (transfusion, feeds, and cares), were electronically annotated in real-time.

### Data processing

Simultaneous StO_2_ and SpO_2_ data were downloaded at a sampling rate of 1000 hertz (Hz) in LabChart reader format (.adicht files) using a PowerLab system [25]. The.adicht files were converted into.mat file format using a simple Python script [26] (Python^TM^ version 3.7.3) and resampled at 1Hz for faster processing. Data that could not be physiologically explained (e.g., the absence of variability, [24] a 30% step change in StO_2_ between two subsequent data points for StO_2_ [27]) were removed. Data during the period of ‘cares’ were presumed to be artefactual because NIRS sensors were lifted for inspection of underlying skin during this period; babies underwent a nappy change, oral care, change of position, etc., during this period that would have caused significant movement artifacts. The removed segments were replaced with ‘NaN’ or ‘Not a Number’ which is recognized by Matlab (MATLAB 9.3, The MathWorks, Inc., Massachusetts, United States) and ignored for all subsequent processing.

**Fig 1:**
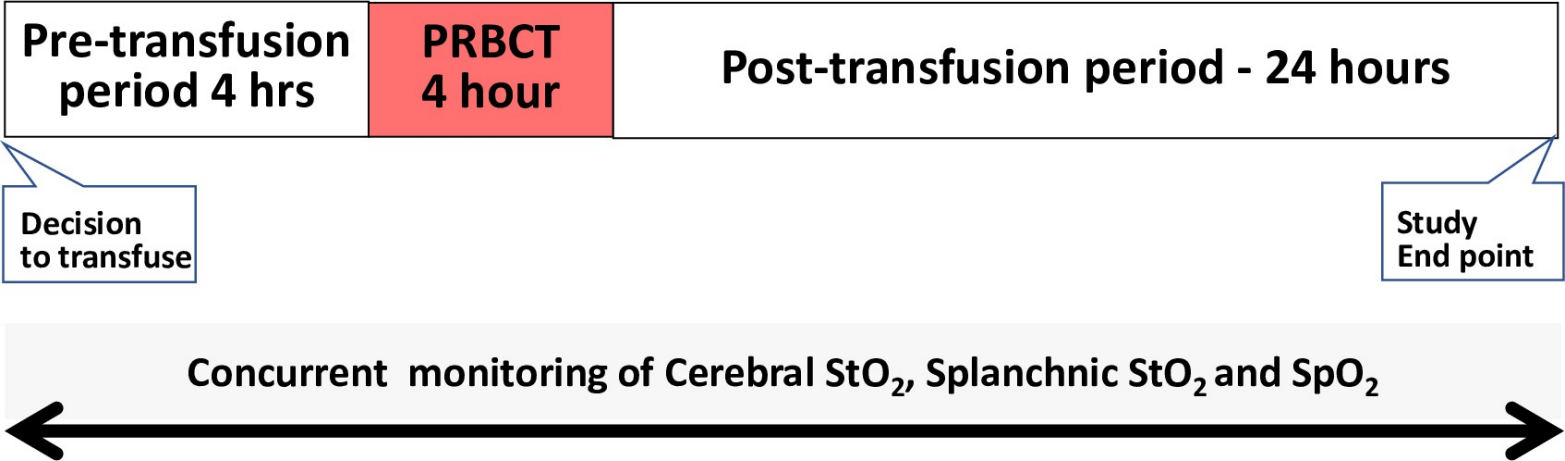
Outline of the study procedure. PRBCT, Packed red blood cell transfusion; SpO_2_, arterial oxygen saturation measured by Pulse Oximetry; StO_2_, tissue oxygen saturation measured by near-infrared spectroscopy; FTOE, Fractional tissue oxygen extraction derived from StO_2_ and SpO_2_.

The study extended from 4 hours before the beginning of PRBCT, 4 hours during PRBCT and up to 24 hours after completion of PRBCT **(Fig 1).** FTOEc and FTOEs were derived from the real-time StO_2_ and SpO_2_ data for each baby. The real-time series of values were used to construct a baseline (mean of the pre-transfusion FTOE over 4 hours before the beginning of PRBCT), and a set of hourly post-baseline mean values (FTOE1-28). The grouping of the real-time values in this way was specified *a priori* as a clinically meaningful approach for facilitating the interpretation of oxygen kinetic changes during and after PRBCT as compared to pre-transfusion values.

### Sample size

A pragmatic sample size of 30 was selected to facilitate reasonable enrolment based on the number of potential eligible infants typically admitted to the neonatal intensive care unit (NICU) and the rate of transfusion.

### Statistical analysis

A Mixed Models for Repeated Measures (MMRM) analysis was applied to the oxygen kinetic data (FTOEc, FTOEs) from all 29 infants. Time point (i.e. baseline, and post-transfusion hours 1 to 28) was fitted as a fixed effect, and the infant was fitted as a random effect. The MMRM was used to perform paired comparisons between the baseline (FTOE0), and each of the post-baseline (FTOE1-28) hourly mean values. Dunnett method was used to adjust for multiplicity. All p-values were two-sided, and values less than 0.05 were considered statistically significant.

## Results

### Demographic features

**Fig 2:**
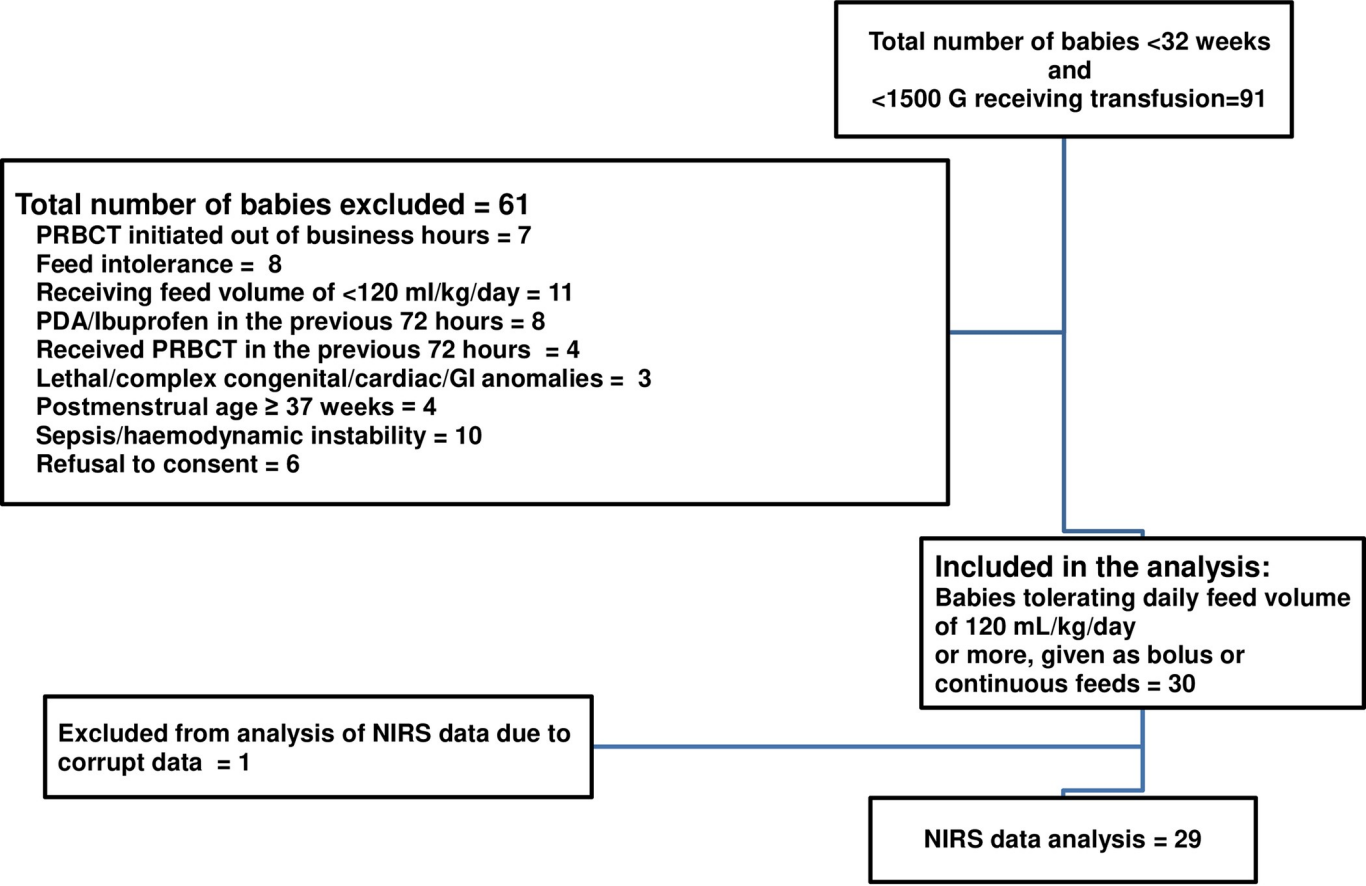
Flow diagram of the study participants. Enrolled and excluded participants are shown in the flow diagram. GI, Gastrointestinal; NIRS, Near-infrared spectroscopy; PDA, patent ductus arteriosus; PRBCT, packed red blood cell transfusion.

Characteristics of potentially eligible, excluded and analyzed babies are depicted in Fig 2. Twenty-nine out of 30 enrolled infants were included in the FTOE analysis, as one baby had corrupted NIRS data that couldn’t be downloaded.

The demography of the study cohort is presented in Table 1.

**Table 1:** Main clinical characteristics of patients (N = 30).

Characteristics	
Gestation, weeks, Median (IQR)	26.4 (25.5–28.1)
Birth weight, Grams, Median (IQR)	923 (655–1064)
Male gender, N. (%)	14 (46.7%)
Restricted umbilical arterial Doppler, N. (%)	5 (16.7%)
Small for Gestation, N. (%)	6 (20%)
Enrolment characteristics	
Postmenstrual age, weeks, Median (IQR)	33.6 (32.4–35)
Postnatal age, days, Median (IQR)	43 (27–59)
Weight, Grams, Median (IQR)	1549 (1113–1882)
Breathing support, N. (%)	
None	5 (16.7%)
Continuous positive airway pressure	15 (50%)
High flow nasal cannula	8 (26.7%)
Low flow oxygen	2 (6.7%)
Daily feed intake, mL/Kg/day, Median (IQR)	155 (150–160)
Formula milk, N. (%)	10 (33.3%)
Caffeine, N. (%)	25 (83.3%)
Patent ductus arteriosus, N. (%)	0 (0%)
Transfusion characteristics	
PRBCT volume (mL/Kg), Median (IQR)	15.3 (15.1–15.4)
Pre transfusion hemoglobin, (g/L), Median (IQR)	93.5 (87.8–102.3)
Post transfusion hemoglobin, (g/L), mean (SD)	124 (17)
Hemoglobin of Packed red cells, (g/L), Median(IQR)	198 (189–203)
Age of packed red cells (days), Median (IQR)	13 (4–24)
Total number of previous transfusions, Median(IQR)	2 (0–5)
Most recent transfusion (days), Median (IQR)	-16 (9–30)
Necrotizing enterocolitis prior to discharge	None
Feed intolerance during the study period	None
Lactate, Mean (SD)	1.7 (0.4)
Heart rate, Mean (SD)	159 (7)
Mean BP, Mean (SD)	48 (7)

### Cerebral and splanchnic FTOE changes in association with PRBCT (Table 2, Fig 3, S1 Table in S1 Appendix)

**Fig 3:**
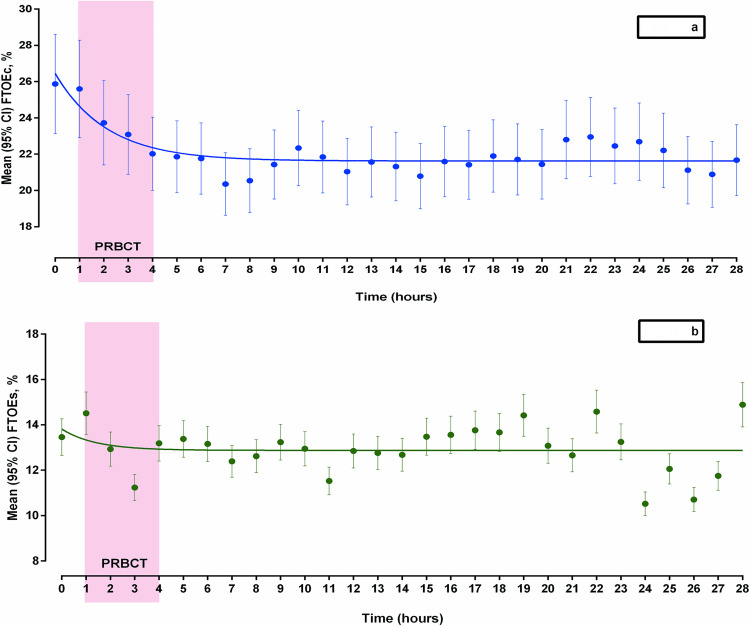
Transfusion-associated changes in cerebral and splanchnic Fractional Tissue Oxygen Extraction (FTOEc and FTOEs) during the study period. **a:** [shows mean (95% CI), and non-linear curve fit for FTOEc]: FTOEc showed a statistically significant drop following Packed Red Blood Cell Transfusion (PBRCT) and remained low, suggesting an improvement in cerebral tissue oxygenation. **b:** [shows mean (95% CI), and non-linear curve fit for FTOEs]: FTOEs showed fluctuations during and after Packed Red Blood Cell Transfusion (PBRCT) without any trend.

FTOEc demonstrated a significant decrease during PRBCT and remained lower than the pre-transfusion level throughout the study period (Fig 3A). Pre-transfusion cerebral FTOE (FTOEc0) was significantly higher (p<0.01) than almost all of the hourly post-transfusion values (except during the first two hours after the beginning of transfusion, FTOEc1, and FTOEc2). FTOEs showed fluctuations throughout the study period without any definite trend (Fig 3B). All of the post-transfusion splanchnic FTOE (FTOEs1-28) were similar to the pre-transfusion value (FTOEs0).

**Table 2:** Exploratory analysis of cerebral and splanchnic fractional tissue oxygen extraction in association with packed red blood cell transfusion—mixed model for repeated measures (n = 29).

Hours	FTOEc, Mean (95% CI), %	Estimated Difference From FTOEc0, %, (95% CI)	P value^a^	FTOEs, Mean (95% CI), %	Estimated Difference From FTOEs0, %, (95% CI)	P value^a^
0	25.87 (23.87 to 27.87)		** **	13.46 (11.04 to 15.88)		
1	25.60 (23.59 to 27.60)	-0.27 (-2.99 to 2.44)	1.00	14.51 (12.09 to 16.93)	1.05 (-3.02 to 5.12)	1.0
2	23.73 (21.72 to 25.73)	-2.14 (-4.86 to 0.57)	0.24	12.93 (10.51 to 15.35)	-0.54 (-4.61 to 3.54)	1.0
3	23.09 (21.09 to 25.10)	-2.77 (-5.49 to -0.06)	0.04	11.24 (8.82 to 13.66)	-2.22 (-6.29 to 1.85)	0.8
4	22.02 (20.02 to 24.02)	-3.85 (-6.56 to -1.13)	0.0006	13.19 (10.77 to 15.61)	-0.27 (-4.34 to 3.80)	1.0
5	21.86 (19.85 to 23.86)	-4.01 (-6.73 to -1.29)	0.0003	13.38 (10.96 to 15.80)	-0.08 (-4.16 to 3.99)	1.0
6	21.76 (19.75 to 23.76)	-4.11 (-6.83 to -1.40)	0.0002	13.16 (10.74 to 15.58)	-0.30 (-4.37 to 3.77)	1.0
7	20.35 (18.35 to 22.35)	-5.52 (-8.23 to -2.80)	<0.0001	12.39 (9.97 to 14.81)	-1.07 (-5.14 to 3.00)	1.0
8	20.54 (18.54 to 22.55)	-5.32 (-8.04 to -2.61)	<0.0001	12.62 (10.20 to 15.04)	-0.84 (-4.91 to 3.23)	1.0
9	21.43 (19.43 to 23.43)	-4.44 (-7.15 to -1.72)	<0.0001	13.24 (10.82 to 15.66)	-0.22 (-4.30 to 3.85)	1.0
10	22.34 (20.34 to 24.34)	-3.53 (-6.24 to -0.81)	0.003	12.95 (10.53 to 15.37)	-0.51 (-4.58 to 3.56)	1.0
11	21.85 (19.85 to 23.85)	-4.02 (-6.74 to -1.30)	0.0003	11.53 (9.11 to 13.95)	-1.93 (-6.00 to 2.14)	0.9
12	21.04 (19.04 to 23.04)	-4.83 (-7.54 to -2.11)	<0.0001	12.85 (10.43 to 15.27)	-0.62 (-4.69 to 3.46)	1.0
13	21.57 (19.57 to 23.57)	-4.30 (-7.02 to -1.58)	<0.0001	12.76 (10.34 to 15.18)	-0.70 (-4.77 to 3.37)	1.0
14	21.32 (19.32 to 23.32)	-4.55 (-7.27 to -1.83)	<0.0001	12.68 (10.26 to 15.10)	-0.79 (-4.86 to 3.29)	1.0
15	20.79 (18.78 to 22.79)	-5.08 (-7.80 to -2.37)	<0.0001	13.48 (11.06 to 15.90)	0.02 (-4.05 to 4.09)	1.0
16	21.59 (19.58 to 23.59)	-4.28 (-7.00 to -1.57)	<0.0001	13.56 (11.14 to 15.98)	0.10 (-3.98 to 4.17)	1.0
17	21.42 (19.42 to 23.43)	-4.44 (-7.16 to -1.73)	<0.0001	13.76 (11.34 to 16.18)	0.30 (-3.78 to 4.37)	1.0
18	21.90 (19.87 to 23.94)	-3.96 (-6.73 to -1.19)	0.0005	13.67 (11.19 to 16.15)	0.21 (-3.94 to 4.36)	1.0
19	21.71 (19.68 to 23.74)	-4.16 (-6.93 to -1.39)	0.0002	14.42 (11.94 to 16.89)	0.95 (-3.20 to 5.10)	1.0
20	21.44 (19.41 to 23.48)	-4.42 (-7.19 to -1.66)	<0.0001	13.08 (10.60 to 15.56)	-0.38 (-4.53 to 3.77)	1.0
21	22.80 (20.75 to 24.85)	-3.07 (-5.87 to -0.27)	0.02	12.66 (10.16 to 15.17)	-0.80 (-4.99 to 3.39)	1.0
22	22.95 (20.90 to 25.00)	-2.92 (-5.71 to -0.12)	0.03	14.58 (12.07 to 17.08)	1.12 (-3.08 to 5.31)	1.0
23	22.45 (20.40 to 24.50)	-3.41 (-6.21 to -0.62)	0.006	13.25 (10.74 to 15.76)	-0.21 (-4.40 to 3.98)	1.0
24	22.69 (20.64 to 24.74)	-3.18 (-5.97 to -0.38)	0.01	10.52 (8.04 to 13.00)	-2.94 (-7.09 to 1.21)	0.4
25	22.21 (20.16 to 24.26)	-3.66 (-6.45 to -0.86)	0.002	12.06 (9.59 to 14.54)	-1.40 (-5.55 to 2.75)	1.0
26	21.12 (19.07 to 23.17)	-4.74 (-7.54 to -1.95)	<0.0001	10.71 (8.23 to 13.18)	-2.76 (-6.91 to 1.39)	0.5
27	20.89 (18.84 to 22.94)	-4.98 (-7.77 to -2.18)	<0.0001	11.75 (9.27 to 14.23)	-1.71 (-5.86 to 2.44)	1.0
28	21.67 (19.64 to 23.71)	-4.19 (-6.96 to -1.43)	0.0002	14.89 (12.42 to 17.37)	1.43 (-2.72 to 5.58)	1.0

FTOEc, Cerebral fractional tissue oxygen extraction; FTOEs, Splanchnic fractional tissue oxygen extraction; FTOE0, Fractional tissue oxygen extraction during the pre-transfusion period. CI, confidence interval

^a^The Dunnett method was used to correct P values and 95% CIs for the pairwise tests.

## Discussion

Our study indicated that cerebral tissue oxygenation promptly improved following PRBCT and remained significantly better for at least 24 hours after its completion. However, splanchnic tissue oxygenation did not change for 24 hours after the completion of PRBCT.

Higher FTOEc during anemia compared to the post-transfusion period indicates that there is cerebral oxygen delivery-consumption mismatch during anemia, which improve following PRBCT. The post-transfusion decrease in FTOEc is likely reflective of an improvement in oxygen delivery to match oxygen consumption. Our results are in alignment with other studies [28–30] and underscore cerebral autoregulatory response to the improvement in oxygen content from PRBCT [31–33].

Our study showed that FTOEs remained unaltered despite PRBCT, for at least 24 hours after the completion of PRBCT. A change in FTOEc but not FTOEs following PRBCT may simply indicate that cerebral vasculature is more responsive to increased oxygenation than splanchnic vasculature. Oxygen consumption of the cerebral tissue is much higher compared to the splanchnic tissue [34]. Our finding most likely reflects the body’s autoregulatory mechanism to fulfill cerebral oxygen consumption delivery balance in preference to that of splanchnic tissue.

Lack of change in splanchnic oxygenation could indicate one of two possibilities: One possibility may be that there was no pre-existing splanchnic oxygen-consumption delivery imbalance during anemia. This may be due to the compensatory increase in splanchnic perfusion in response to normovolemic haemodilution (low haematocrit) during anemia, as shown in animal studies [35]. However, this explanation is unlikely to justify our findings. This is because the compensatory response is not unique to splanchnic circulation and is seen even in the cerebral circulation. Preterm human and animal studies have shown an inverse correlation of haematocrit with cerebral blood flow [36, 37]. As a result, viscosity is unlikely to explain the differential behaviour of cerebral and splanchnic perfusion. Moreover, the assumption that splanchnic oxygen demand was met during the anemic phase and thus there was no physiologic reason to change FTOEs after transfusion cannot explain why cerebral oxygenation should improve following a PRBCT. We believe that the second possibility is a physiologically more plausible explanation of our findings. It is likely that there was splanchnic oxygen consumption-delivery imbalance during the anemic phase due to preferential cerebral oxygenation. This is supported by studies which demonstrated that anemia-induced tissue hypoxia is heterogeneous involving renal and splanchnic before cerebral tissue [38, 39]. We propose that the splanchnic oxygen consumption-delivery imbalance continued during the early post-PRBCT phase due to preferential improvement in cerebral oxygenation during this phase. In our study, FTOEs did not improve during the 24 hours period of monitoring. However, in another study by White et al. [40]. FTOEs improvement commenced only after 36 hours post-PRBCT. Although we did not perform an extended period of monitoring beyond 24 hours, it is logical to propose a physiologically plausible hypothesis that there was perhaps a delay in recovery of splanchnic oxygen consumption-delivery balance consequent to preferential cerebral oxygenation in the early post-PRBCT phase. The physiologic buffer to secure cerebral oxygenation, with the trade-off of continued splanchnic tissue hypoxia in the immediate post-transfusion period may be a factor in the pathogenesis of TANEC. Splanchnic susceptibility may be further exacerbated by other factors, including storage RBC lesion that prevents effective oxygen offloading [41] and the negative effect of continued feeding during and immediately after PRBCT [23, 42–45]. The median (IQR) age of transfused packed red blood cells was 13 (4–24) days. All of our babies were fed before, during and after PRBCT.

Other studies have demonstrated variable results of splanchnic oxygenation, some showing improvement [30, 46–48] while others showing deterioration during [49] and after PRBCT [50]. A brief summary of these studies is described in **S2 Table in S1 Appendix**. Previous studies are comparable with the similar number of participants, gestational age, enrolment weight and postnatal age, as well as PRBCT doses. The exception here is Bronshtein et al. with only 5 infants. However there are a number of striking methodological differences from our study. Unlike our cohort, feeds were completely withheld [46, 48, 50], minimal [47, 51], or feeding status not mentioned [30, 49]. While splanchnic oxygenation has been shown to improve in studies where feeds were withheld [13] the results are contradictory in studies where feeds were given during transfusion, with some studies reporting no change [52] and others showing a decline [44, 45]. In contrast, in our study, all infants were on full enteral feeds before, during, and after PRBCT. Hence lack of improvement of splanchnic perfusion can be explained by the associated negative effects of feeding on splanchnic perfusion. Human and animal studies [42–44] suggest that splanchnic tissue oxygenation is negatively associated with feeding in those who received PRBCT. Another likely reason for our results to be different from other studies include methodological differences in NIRS monitoring. Unlike FTOEc, the FTOEs is inherently associated with wider confidence intervals [17, 21, 22], momentary losses of NIRS signals, and periods of very low splanchnic StO_2_ readings [17, 53]. A physiological reason for this is the active peristalsis of healthy bowel resulting in variable segments of the intestine being interrogated, as well as the changing gas-fluid-faecal interfaces [54]. The reliability of splanchnic tissue oxygenation measurements are likely to be compromised by artifacts picked up by episodic and short duration of NIRS monitoring [55]. Mintzer et al. [22] recommended utilizing relatively short periods as a preferred data averaging interval and using these epochs to evaluate trends in splanchnic tissue oxygenation over longer periods. Thus, splanchnic tissue oxygenation is meaningful only if monitored continuously, at high sampling frequency, and over longer periods. Accordingly, our approach towards methodology including splanchnic oxygenation monitoring, data extraction, and analysis is superior to many other published studies. Unlike other studies [46–49] where the sampling frequency varied from one per 6 sec to 1 minute, our sampling frequency was 1 per second. A more frequent sampling is likely to pick up inherent changes compared to less frequent sampling. We performed continuous rather than episodic monitoring so as to not miss out on changes occurring at other times in the specified period of monitoring. Many studies, on the other hand, performed episodic measurements varying from spot measurement [46] to 20 minutes [47–49]. Even though some studies performed a longer duration of monitoring over 4 to 11 hours [30, 50], they employed longer data averaging period, and used a single mean ± SD value over the entire period of 4–11 hours, which obviously obscures the variability in NIRS recordings during this period. We calculated mean ± SD every hour during and after PRBCT to determine how splanchnic oxygenation varied during this period. Therefore our approach has more adequately captured splanchnic variability, and overcome caveats associated with monitoring of splanchnic oxygenation. Finally, our statistical approach is more robust compared to previous studies which used either a repeated measures ANOVA or t-test with or without correction for the multiplicity of p values [30, 46–50]. We performed a Mixed Models for Repeated Measures (MMRM) analysis. Due to their ability to consider both fixed and random effects, mixed models are ideally suited in this setting to assess the trajectory of the outcome. Unlike other methods, MMRM has an additional advantage of accommodating unbalanced data patterns due to random missing of data and are associated with lesser inflation of type 1 error [56–58]. We also used Dunnett’s method to adjust for multiplicity of 2-sided p-values.

Another unique strength of our study is the simultaneous monitoring of cerebral and splanchnic tissue oxygenation in association with PRBCT. There are only a few other studies where simultaneous monitoring was performed [30, 48]. However, these studies have applied a problematic methodological approach. Either splanchnic oxygenation was monitored episodically (short periods), or very long periods of data averaging (as long as 4–11 hrs) were used, or very short segments of data were analysed in these studies.

There are currently no widely published normative data for splanchnic oxygenation [59]. The existing studies are limited by the small number of enrolled candidates [17, 24, 60]. As a result, it is recommended that one should follow a trend from the baseline values rather than relying on absolute values [61, 62]. As suggested by Bailey and Mally [61] taking the two trends of cerebral and splanchnic oxygenation and comparing them with each other in an individual patient, may account for more than just a trend and it could quite possibly reveal “absolutely” about what is happening clinically to that particular patient. As a result, splanchnic tissue oxygenation is meaningful only if monitored continuously and over longer periods as compared to cerebral tissue oxygenation. This is one of the greatest strengths of our study.

We acknowledge several limitations of the study. Our study describes the autoregulation of cerebral circulation as compared to splanchnic circulation when hypoxia is reversed by PRBCT. Our findings, along with many others [28–30], indicate that there is a statistically significant improvement in FTOEc, while FTOEs remained unaltered. Although improvement in FTOEc is statistically significant, its biological significance is unknown. Substantial variability exists in NICU transfusion practices, with liberal vs. restriction transfusion guidelines [63]. These variations reflect the long-established controversies regarding neurological outcomes following restrictive versus liberal transfusion thresholds. Although some of the earlier studies favored liberal transfusion practice [64, 65], it is becoming more apparent, especially with results from the most two recent randomized controlled trials [66, 67], that there is no difference in survival without neurodevelopmental impairment at 24 months between extremely low birth weight infants transfused at higher versus lower haemoglobin thresholds. Hence the statistically significant improvement of cerebral oxygenation should be interpreted cautiously in a clinical context, and PRBCT should not be considered as an effective intervention to prevent poor neurodevelopmental outcomes.

Furthermore, FTOE is confounded by the change in viscosity during anemia vs. post-PRBCT period, with viscosity impacting microvascular oxygenation. Anemia has a dual effect on tissue oxygenation. On the one hand, it decreases the oxygen-carrying capacity of the blood. On the contrary, it is associated with decreased viscosity, which in turn improves oxygen delivery. Hence microvascular oxygenation is potentially at its best when the haematocrit-to-viscosity ratio (HVR) is highest. Using NIRS, hematocrit-to-viscosity ratios have been positively correlated with cerebral StO_2_ [68]. A further limitation of our study is that viscosity was not measured for practical reasons as continuous measurements are not available, and frequent interval sampling is too invasive and hence ethically not justifiable.

Only hemodynamically stable infants were included due to concerns that hemodynamic instability may confound splanchnic perfusion. These exclusion criteria may explain why none of our babies developed TANEC. The generalizability of our findings may also be limited by the small sample size. Moreover, we cannot exclude bias due to the selective enrolment of babies during office hours. The study involved stable premature babies with postmenstrual age 33.6 (32.4–35) weeks, and as such, caution must be exercised in interpreting the results of the study in more premature infants with cardiorespiratory instability. The study did not involve controls who did not receive PRBCT. However, the aim of the study was to compare the changes of tissue oxygenation in the two different organs of the body when simultaneously exposed to PRBCT. The behavior of tissue oxygenation of the vital organ (brain) on exposure to PRBCT was compared to the non-vital organ (gut) on exposure to the same intervention under exactly the same condition. The design of the study allowed one organ to act as a control against the other. The findings of the study suggest that brain oxygenation behaves completely differently from splanchnic tissue oxygenation on exposure to PRBCT. As this is a comparison of tissue oxygenation following PRBCT, between two organs, the study design allows us to answer the research question without using controls. Due to the observational nature of the study, an association rather than a cause-effect relation can be ascertained between PRBCT and regional oxygenation.

Nevertheless, the strength of this study is concurrently investigating the association of PRBCT with both splanchnic and cerebral tissue oxygenation through the continuous 32 hours of monitoring of high voluminous data points, as against the shorter duration and episodic nature of monitoring in other studies [28, 30]. Rigorous methodology and high-quality statistical analysis add to the strength. A particular contribution of the study is the use of a prospective cohort design to validate existing literature. Unlike many others, we measured FTOE which gives a better understanding of oxygen consumption–delivery coupling rather than simply the tissue oxygen levels (StO_2_).

In summary, we have elucidated a possible physiological mechanism of PRBCT-associated regional tissue oxygenation changes. Our hierarchical concept of differential improvement in regional tissue oxygenation provides a possible mechanistic basis for the continued vulnerability of the gut to hypoxic damage in the immediate post-transfusion period that may contribute to the development of TANEC.

## Supporting information

S1 Appendix (DOCX)
