# Supplementary material for: Hierarchical improvement of regional tissue oxygenation after packed red blood cell transfusion
Source: PLoS One. 2022 Jul 20;17(7):e0271563. doi: 10.1371/journal.pone.0271563 (PMC9299358; doi:10.1371/journal.pone.0271563)
Supplement: S1 Appendix — (DOCX) [file pone.0271563.s001.docx]

**S1 Table: Cerebral and splanchnic oxygen kinetics in association with transfusion**

|  | **hour** | **Cerebral StO_2_** | **Splanchnic StO_2_** | **SpO_2_** | **Cerebral FTOE** | **Splanchnic FTOE** |
| --- | --- | --- | --- | --- | --- | --- |
| Before PRBCT | 0 | [29] 71.45 (5.55) | [29] 83.38 (7.56) | [29] 96.38 (2.44) | [29] 25.87 (5.42) | [29] 13.46 (8.03) |
| During PRBCT | 1 | [29] 71.38 (5.86) | [29] 81.97 (6.54) | [29] 95.94 (3.98) | [29] 25.60 (5.38) | [29] 14.51 (7.04) |
|  | 2 | [29] 72.95 (5.46) | [29] 83.56 (7.24) | [29] 95.68 (3.82) | [29] 23.73 (5.14) | [29] 12.93 (7.45) |
|  | 3 | [29] 73.07 (6.22) | [29] 85.40 (5.89) | [29] 95.12 (4.63) | [29] 23.09 (6.69) | [29] 11.24 (5.54) |
|  | 4 | [29] 74.42 (5.13) | [29] 82.99 (5.73) | [29] 95.43 (3.28) | [29] 22.02 (4.60) | [29] 13.19 (5.40) |
| After PRBCT | 5 | [29] 75.57 (4.19) | [29] 83.79 (5.70) | [29] 96.73 (1.93) | [29] 21.86 (4.41) | [29] 13.38 (5.57) |
|  | 6 | [29] 74.53 (4.59) | [29] 83.08 (6.16) | [29] 95.29 (3.83) | [29] 21.76 (4.28) | [29] 13.16 (5.28) |
|  | 7 | [29] 76.35 (5.07) | [29] 84.07 (5.85) | [29] 95.84 (2.53) | [29] 20.35 (4.58) | [29] 12.39 (6.30) |
|  | 8 | [29] 75.98 (3.75) | [29] 83.58 (6.42) | [29] 95.65 (2.85) | [29] 20.54 (3.48) | [29] 12.62 (6.25) |
|  | 9 | [29] 75.24 (4.42) | [29] 83.24 (6.31) | [29] 95.81 (3.30) | [29] 21.43 (4.40) | [29] 13.24 (6.33) |
|  | 10 | [29] 74.80 (4.03) | [29] 83.86 (6.51) | [29] 96.31 (2.20) | [29] 22.34 (3.82) | [29] 12.95 (6.27) |
|  | 11 | [29] 74.56 (4.81) | [29] 84.47 (4.60) | [29] 95.42 (3.53) | [29] 21.85 (4.28) | [29] 11.53 (4.80) |
|  | 12 | [29] 75.14 (4.43) | [29] 83.61 (6.05) | [29] 93.84 (7.18) | [29] 21.04 (4.84) | [29] 12.85 (5.62) |
|  | 13 | [29] 75.32 (4.80) | [29] 83.79 (4.90) | [29] 96.08 (2.56) | [29] 21.57 (5.14) | [29] 12.76 (5.16) |
|  | 14 | [29] 75.63 (4.51) | [29] 83.97 (6.14) | [29] 96.15 (2.31) | [29] 21.32 (4.69) | [29] 12.68 (5.90) |
|  | 15 | [29] 74.88 (6.26) | [29] 81.98 (6.24) | [29] 94.56 (5.04) | [29] 20.79 (5.51) | [29] 13.48 (6.33) |
|  | 16 | [29] 74.66 (4.94) | [29] 82.32 (6.64) | [29] 95.27 (3.25) | [29] 21.59 (5.32) | [29] 13.56 (6.87) |
|  | 17 | [29] 75.25 (4.87) | [29] 82.59 (6.26) | [29] 95.79 (2.42) | [29] 21.42 (4.95) | [29] 13.76 (6.46) |
|  | 18 | [27] 74.92 (5.12) | [27] 82.62 (7.37) | [27] 95.75 (2.49) | [27] 21.76 (5.02) | [27] 13.75 (7.11) |
|  | 19 | [27] 74.87 (6.22) | [27] 81.60 (7.32) | [27] 95.43 (2.92) | [27] 21.56 (5.86) | [27] 14.49 (7.32) |
|  | 20 | [27] 75.37 (5.49) | [27] 83.13 (6.82) | [27] 95.76 (2.24) | [27] 21.29 (5.42) | [27] 13.16 (7.20) |
|  | 21 | [27] 74.13 (5.39) | [27] 83.90 (6.16) | [27] 94.96 (5.25) | [26] 22.98 (5.31) | [26] 12.65 (6.60) |
|  | 22 | [27] 73.92 (7.10) | [27] 82.17 (7.96) | [27] 94.93 (5.29) | [26] 23.14 (7.54) | [26] 14.57 (8.34) |
|  | 23 | [27] 74.31 (7.16) | [27] 83.41 (9.02) | [27] 94.79 (4.58) | [26] 22.64 (7.15) | [26] 13.24 (8.88) |
|  | 24 | [27] 74.04 (7.83) | [27] 85.38 (6.18) | [27] 94.77 (4.59) | [26] 22.87 (7.74) | [27] 10.60 (6.08) |
|  | 25 | [27] 74.79 (6.33) | [27] 83.53 (6.23) | [27] 95.16 (3.83) | [26] 22.39 (6.03) | [27] 12.14 (6.59) |
|  | 26 | [27] 75.67 (5.67) | [27] 85.37 (5.85) | [27] 95.16 (3.91) | [26] 21.31 (5.91) | [27] 10.78 (6.40) |
|  | 27 | [27] 75.61 (5.34) | [27] 83.91 (5.77) | [27] 94.99 (4.10) | [26] 21.07 (5.27) | [27] 11.83 (6.30) |
|  | 28 | [27] 74.93 (5.94) | [27] 81.70 (8.37) | [27] 95.51 (3.84) | [27] 21.52 (7.52) | [27] 14.97 (8.25) |

**S1 table legend:** Data are represented as [N] Mean (SD); SpO_2_= Arterial oxygen saturation measured by pulse oximeter; StO_2_ = tissue oxygen saturation measured by near-infrared spectroscopy; FTOE = fractional tissue oxygen extraction; PRBCT = Packed red blood cell transfusion.

**S2Table: Studies where splanchnic oxygenation changes differ from our study**

| **Study** | **Bailey et al.[1]** | **Sandal et al.[2]** | **Weaver et al.[3]** | **Banerjee et al.[4]** | **Miller et al.[5]** | **Bronshtein et al.[6]** | **Mintzer et al.[7]** |
| --- | --- | --- | --- | --- | --- | --- | --- |
| **Number of babies receiving PRBCT** | 30 | 23 | 35 | 20 in each group, based on postnatal age: <1 week; 1-4 weeks; >4 weeks | 30 | 5 | 10 |
| **Gestation**  **(weeks)** | Mean ± SD  28.4 ± 3 | Mean ± SD  27.7 ± 1.8 | n/a | Median (range)  Gr 1: 26 (23-27)  Gr 2: 25 (23-30)  Gr 3: 26 (24-34) | Mean ± SD  25.5 ± 2.1 | Mean ± SD  28.6 ± 3.6 | Mean ± SD  26 ± 0 |
| **Birth Weight**  **(grams)** | Mean ± SD  1115 ± 426 | Mean ± SD  990 ± 285 | Mean ± SD  1305 ± 400 | Median (range)  Gr 1:763  (600-1180)  Gr 2:740  (600-1240)  Gr 3:793  (520-1746) | Mean ± SD  768.43 ± 241.87 | Mean ± SD  1159 ± 409 | Mean ± SD  879 ± 49 |
| **Weight at enrollment / transfusion**  **(gram)** | Mean ± SD  1415 ± 456 | Mean ± SD  1416 ± 357 | Mean ± SD  1615 ± 323 | Median (range)  Gr 1:774  (700-1180)  Gr 2:805  (680-1250)  Gr3:1125  (887-2045) | n/a | n/a | Mean ± SD  855 ± 38 |
| **Postnatal age at enrollment (days)** | Not available  PMA was  Mean±SD  32.9 ± 3.4 | Mean ± SD  45 ± 14.3 | Mean ± SD  25.4 ± 16.9 | Median (range)  Gr 1: 5 (1-7)  Gr 2: 14 (8-27)  Gr 3: 45(29-93) | Mean ± SD  13.9 ± 11.78 | Mean ± SD  44 ± 29 | Mean ± SD  3 ± 0 |
| **PRBCT dose** | 15 mL/kg over 4 hrs | 15 mL/kg over 2-4 hrs | 10-20 mL/kg over 3-4 hours | 15 ml/Kg over 3 hours | 10-20 mL/kg, variable duration | Dose unspecified, over 4 hrs | 15 mL/kg over 3-4 hours |
| **Mean ± SD**  **Hb (G/dL)**  **/Hct (%)** | 9.3 ± 1.2 | 8.7 ± 2.3 | 8.0 ± 0.3 | Gr1 <12  Gr 2< 10  Gr 3 < 8 | Not recorded | Hematocrit 27.8 ± 6 | Hematocrit  35.2 ± 1.2 |
| **Feeds** | Withheld during PRBCT | Not available | Withheld before, during and after PRBCT | Not all babies were receiving full feeds | Withheld during and after PRBCT | Not available | Not on full feeds |
| **NIRS monitoring duration** | 20 minutes epochs of StO_2_s monitoring before, during, and after PRBCT | 10 -11 hours before PRBCT, during PRBCT, and 10-11 hours after PRBCT | Spot FTOE measurements once every 30 minutes for 3 hours. No post-transfusion measurement. | Continuous monitoring from 15-20 minutes before the commencement of PRBCT until 15-20 minutes after its completion. | Minimum 2 hours before PRBCT, during PRBCT, and 4 hours after PRBCT | five-minute time epochs at 7 different time points: at 1 hr before, at the beginning, hrly for 4 hrs during and 1 hr after PRBCT | 1 hour before PRBCT, duration of PRBCT, 2 hours after PRBCT. |
| **Frequency of NIRS sampling** | Every 30 seconds | Every one minute | Not mentioned | Every 6 sec | Every 30 sec | Every 1 minute | Every 30 sec |
| **Statistical approach** | Mean ± SD before, during, and after (immediately and at 12 hours) PRBCT were compared by one-way repeated measures ANOVA and Turkey’s HSD test | Mean ± SD before, during, and after PRBCT were compared by t-test | Mean ± SD between these measurements were compared by ANOVA | Mean ± SD of 15-20 minute recordings before PRBCT, 1 hr into PRBCT, 2 hrs into PRBCT, and post-PRBCT calculated. Repeated measures ANOVA between these mean values, with Bonferroni correction. | Mean ± SD of the recording before PRBCT was compared with the Mean ± SD of the reading during PRBCT and after PRBCT using ANOVA | Mean ± SD at 7 different epochs were compared by Repeated measures ANOVA | Repeated measures ANOVA |
| **Results** | Significant improvement in mean StO_2_s during and after PRBCT (p=0.05) | Significant improvement in mean FTOEs (p=0.07) | Significant improvement in mean FTOEs (p<0.05) | Significant improvement in StO_2_s and FTOEs (p<0.05) | Significant improvement in StO_2_s during PRBCT from 40.3% to 41.5, (P < 0.001) but significant decrease to 34.9% in the post PRBCT period (P < 0.001). | Significant decrease in tissue oxygen saturation during PRBCT (at 2^nd^, 3^rd^, and 4^th^ hour), followed by recovery at the end of PRBCT. | Significant improvement in splanchnic oxygenation post PRBCT |

**S2 table legend:** SpO_2_= Arterial oxygen saturation measured by pulse oximeter; StO_2 =_ tissue oxygen saturation measured by near-infrared spectroscopy; FTOE = fractional tissue oxygen extraction; FTOEs = Splanchnic fractional tissue oxygen extraction; FTOEc = Cerebral fractional tissue oxygen extraction; PRBCT=Packed red blood cell transfusion; Hb = Hemoglobin; Hct = Hematocrit; NIRS = Near-infrared spectroscopy.

1. Bailey SM, Hendricks-Muñoz KD, Wells JT, Mally P. Packed red blood cell transfusion increases regional cerebral and splanchnic tissue oxygen saturation in anemic symptomatic preterm infants. *American journal of perinatology.* 2010;27(06):445-453.

2. Sandal G, Oguz SS, Erdeve O, Akar M, Uras N, Dilmen U. Assessment of red blood cell transfusion and transfusion duration on cerebral and mesenteric oxygenation using near‐infrared spectroscopy in preterm infants with symptomatic anemia. *Transfusion.* 2014;54(4):1100-1105.

3. Weaver B, Guerreso K, Conner EA, Russell K, Vogel R, Rodriguez M. Hemodynamics and Perfusion in Premature Infants During Transfusion. *AACN advanced critical care.* 2018;29(2):126-137.

4. Banerjee J, Leung T, Aladangady N. Blood transfusion in preterm infants improves intestinal tissue oxygenation without alteration in blood flow. *Vox sanguinis.* 2016;111(4):399-408.

5. Miller HD, Penoyer DA, Baumann K, et al. Assessment of mesenteric tissue saturation, oxygen saturation, and heart rate pre-and post-blood transfusion in very low-birth-weight infants using abdominal site near-infrared spectroscopy. *Advances in Neonatal Care.* 2017;17(5):E3-E9.

6. Bronshtein V, LaGamma E, Curry J, Hoffman JG, Parvez B. Visual light spectrography (VLS) for detecting alteration in tissue oxygenation with administration of packed red blood cells (PRBC) in very low birth weight (VLBW) premature infants. *Neonatology Today.* 2009;4(02):1-6.

7. Mintzer J, Parvez B, Chelala M, Alpan G, LaGamma E. Monitoring regional tissue oxygen extraction in neonates< 1250 g helps identify transfusion thresholds independent of hematocrit. *Journal of neonatal-perinatal medicine.* 2014;7(2):89-100.
